# Clinical characteristics of persistent postural‐perceptual dizziness and its visual subtype in Korean patients: A multicenter cross‐sectional study

**DOI:** 10.1002/brb3.3389

**Published:** 2024-01-31

**Authors:** Joo Hyun Park, Thanh Tin Nguyen, Sung‐Hee Kim, Ji‐Yun Park, Seunghee Na, Eun‐Ju Jeon, Ji won Seo, Chang Gun Cho, Se‐Joon Oh, Sung‐Won Choi, Kwang‐Dong Choi, Seo‐Young Choi, Ji Eun Choi, Sung‐Kwang Hong, Won‐Ho Chung, Young Sang Cho, Hwan Ho Lee, Yong‐Hwi An, Kyu‐Hee Han, Hyung Lee, Hyun Ah Kim, Ho Yun Lee, Jong‐Dae Lee, Se A Lee, Sun‐Young Oh

**Affiliations:** ^1^ Department of Otorhinolaryngology‐Head and Neck Surgery Dongguk University Ilsan hospital Goyang South Korea; ^2^ Department of Neurology Jeonbuk National University Hospital, Jeonbuk National University School of Medicine Jeonju South Korea; ^3^ Department of Neurology Ewha Womans University Mokdong Hospital Seoul South Korea; ^4^ Department of Neurology Ulsan University Hospital Ulsan South Korea; ^5^ Department of Neurology The Catholic University, Incheon Saint Mary's Hospital Incheon South Korea; ^6^ Department of Otorhinolaryngology‐Head and Neck Surgery The Catholic University, Incheon Saint Mary's Hospital Incheon South Korea; ^7^ Department of Otorhinolaryngology‐Head and Neck Surgery Sungkyunkwan University, Samsung Changwon Hospital Changwon South Korea; ^8^ Department of Otorhinolaryngology‐Head and Neck Surgery Pusan National University Hospital Busan South Korea; ^9^ Department of Neurology Pusan National University Hospital Busan South Korea; ^10^ Department of Otorhinolaryngology‐Head and Neck Surgery Dankook University Hospital Cheonan South Korea; ^11^ Department of Otorhinolaryngology‐Head and Neck Surgery Hallym University Sacred Heart Hospital Anyang South Korea; ^12^ Department of Otorhinolaryngology‐Head and Neck Surgery Sungkyunkwan University, Samsung Seoul Hospital Seoul South Korea; ^13^ Department of Otolaryngology‐Head and Neck Surgery Kosin University Hospital Busan South Korea; ^14^ Department of Otorhinolaryngology‐Head and Neck Surgery Eulji University, Nowon Eulji Medical Center Seoul South Korea; ^15^ Department of Otorhinolaryngology‐Head and Neck Surgery National Medical Center Seoul South Korea; ^16^ Department of Neurology Keimyung University Keimyung University Dongsan Hospital Daegu South Korea; ^17^ Department of Otorhinolaryngology‐Head and Neck Surgery Ewha Womans University Mokdong Hospital Seoul South Korea; ^18^ Department of Otorhinolaryngology‐Head and Neck Surgery Soonchunhyang University Bucheon Hospital Bucheon South Korea; ^19^ Department of Pharmacology Hue University of Medicine and Pharmacy Hue University Hue Vietnam

**Keywords:** chronic dizziness, persistent postural‐perceptual dizziness, variant, visual subtype

## Abstract

**Objectives:**

Persistent postural‐perceptual dizziness (PPPD) is a chronic functional vestibular disorder for which the Bárány Society has established diagnostic criteria. This nationwide multicenter study aims to investigate the clinical features of individuals with definite PPPD and clinical variant PPPD who do not fully meet the diagnostic criteria, with a particular focus on visual exaggeration.

**Methods:**

Between September 2020 and September 2021, a total of 76 individuals with definite PPPD and 109 individuals with clinical variant PPPD who did not meet all three exacerbating factors outlined in Criterion B were recruited from 18 medical centers in South Korea. The study gathered information on demographic factors, clinical manifestations, balance scales, and personality assessments.

**Results:**

Comparative analysis between groups with definite PPPD and clinical variant with visual exacerbation revealed no significant differences in sociodemographic characteristics, clinical course, dizziness impact, and specific precipitants. Only disease duration was significantly longer in definite PPPD compared with variant with visual exacerbation. However, the variant without visual exacerbation displayed significantly reduced rates of panic disorder, diminished space‐motion discomfort, lesser impact of dizziness, and decreased prevalence of depression when compared with the definitive PPPD.

**Conclusion:**

This is the first comprehensive nationwide study examining clinical features of both definite PPPD patients and its clinical variants, considering visual exacerbating factors. Differences in dizziness and personality traits emerged between definite PPPD and its potential variant without visual issues. Our results highlight the possibility of a distinct clinical variant of PPPD influenced by visual dependency.

## Graphical abstract

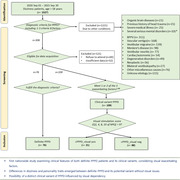

## 1 INTRODUCTION

Persistent postural‐perceptual dizziness (PPPD) is a chronic vestibular syndrome characterized by dizziness or unsteadiness that lasts for at least 3 months (Das et al., 2023; Dieterich & Staab, 2017; Popkirov, Staab, et al., 2018; Popkirov, Stone, et al., 2018; Staab et al., 2017). It is the result of maladaptive dysfunction in balance control and vestibular processing with precipitating events significantly influencing its development (Das et al., 2023; Dieterich & Staab, 2017; Holle et al., 2015; Popkirov, Staab, et al., 2018; Popkirov, Stone, et al., 2018; Staab et al., 2017). Patients with PPPD tend to rely excessively on visual inputs instead of vestibular and somatosensory/proprioceptive inputs, leading to a misjudging sensory outcomes from their actions, which may account for their ongoing dizziness and balance problems (Cousins et al., 2014, 2017; Dieterich & Staab, 2017; Holle et al., 2015; Popkirov, Staab, et al., 2018; Staab, 2020; Staab et al., 2017).

The Bárány Society criteria (Table 1) set the official diagnostic criteria for PPPD in 2017 (Popkirov, Staab, et al., 2018; Popkirov, Stone, et al., 2018; Staab et al., 2017). The importance of exacerbating factors, such as upright posture, active or passive motion, complex visual patterns, and self or environmental motion, is highlighted in related conditions like phobic postural vertigo, space‐motion discomfort, visual vertigo, and chronic subjective dizziness, respectively. These four diseases share clinical features that form the basis of the diagnostic criteria of PPPD. The classification of PPPD remains a matter of debate—whether it is a unique disorder or a compilation of the aforementioned conditions. It is possible to further subdivide PPPD based on the unique characteristics of each exacerbating factor, leading to subtypes such as those dominated by postural, movement, or visual provocation. A recent study utilizing the Niigata PPPD Questionnaire (NPQ) aimed to subtype PPPD based on exacerbating factors (Yagi et al., 2021). Results showed visual stimuli caused 47.4% of the variance, walking/active motion 12%, and passive motion/standing 7.67%. Furthermore, the concept of probable or subthreshold PPPD, which refers to patients not meeting all five of the diagnostic criteria, was found to be lacking sufficient published data to establish a clinically meaningful definition at that time (Adamec et al., 2020; Li et al., 2021; Powell et al., 2020; Staab, 2020; Staab et al., 2017; Trinidade et al., 2023; Wang et al., 2021; Zachou & Anagnostou, 2020). In this study, patients not meeting one or two of Criterion B's factors were classified as a clinical variant PPPD.

**TABLE 1:** Diagnostic criteria for persistent postural‐perceptual dizziness (Bárány Society, 2017).

A. One or more symptoms of dizziness, unsteadiness, or non‐spinning vertigo are present on most days for 3 months or more
1. Symptoms last for prolonged (hours long) periods of time but may wax and wane in severity
2. Symptoms need not be present continuously throughout the entire day
B. Persistent symptoms occur without specific provocation but are exacerbated by three factors
1. Upright posture
2. Active or passive motion without regard to direction or position
3. Exposure to moving visual stimuli or complex visual patterns
C. The disorder is precipitated by conditions that cause vertigo, unsteadiness, dizziness, or problems with balance including acute, episodic, or chronic vestibular syndromes, other neurologic or medical illnesses, or psychological distress
1. When the precipitant is an acute or episodic condition, symptoms settle into the pattern of Criterion A as the precipitant resolves, but they may occur intermittently at first and then consolidate into a persistent course
2. When the precipitant is a chronic syndrome, symptoms may develop slowly at first and worsen gradually
D. Symptoms cause significant distress or functional impairment
E. Symptoms are not better accounted for by another disease or disorder

Although PPPD is acknowledged as a prevalent cause of persistent dizziness in Korea (Dieterich & Staab, 2017; Dieterich et al., 2016; Kim et al., 2020; Powell et al., 2020), there is a lack of research into the clinical characteristics of Korean PPPD patients. In addition, there is insufficient prior evidence determining the prevalence of clinical variants of PPPD that do not meet all three exacerbating criteria, as well as their potential clinical differences when compared to the established PPPD diagnosis. The main aim of this nationwide multicenter study was to investigate the clinical characteristics of patients with definite PPPD and its variants who do not fully meet the diagnostic criteria, with a particular focus on visual exacerbation within the Korean population.

## 2 MATERIALS AND METHODS

### 2.1 Participants

**FIGURE 1:**
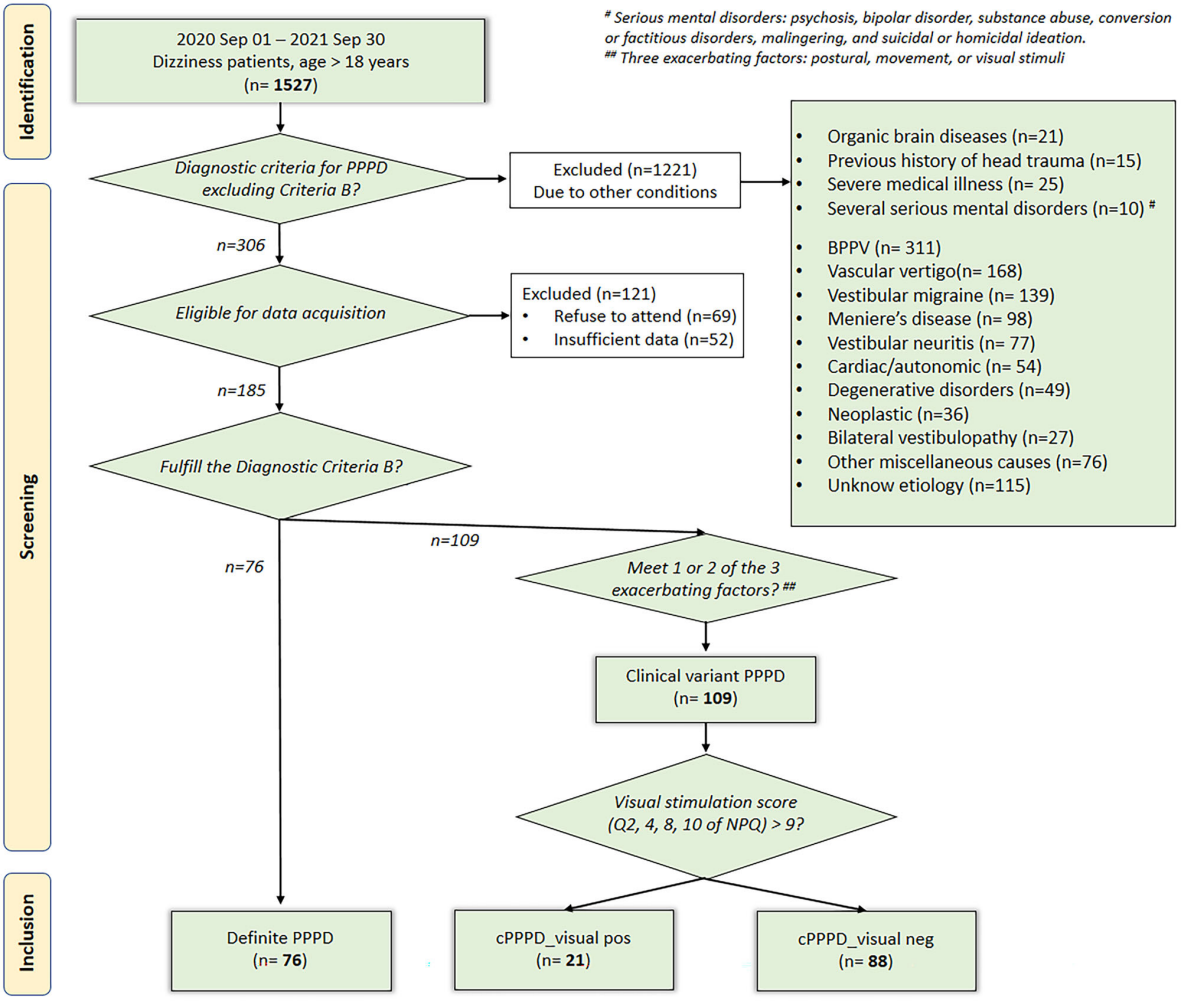
Flow chart represents the data acquisition.

From September 2020 to September 2021, patients were consecutively enrolled from 16 referral centers in Korea (Figure 1). Definite PPPD was diagnosed in adults (>18 years old) by 25 senior neurotologists using the Bárány Society diagnostic criteria and the published description of the disorder (Table 1) (Staab et al., 2017). In the current study, we also include patients meeting all diagnostic criteria except for Criterion B, which specifies symptoms being triggered by one or two exacerbating factors, were classified as the clinical variant of PPPD (Figure 1). This clinical variant was further subdivided into cPPPD_visual pos (exacerbated by visual stimuli) and cPPPD_visual neg (not exacerbated by visual stimuli) based on the NPQ, specifically questions 2, 4, 8, and 10, using a cutoff value of 9 from a total score of 24, with a sensitivity of 82% and specificity of 74% (Yagi et al., 2019, 2021). Exclusion criteria comprised active neuro‐otologic disorders, organic brain diseases, previous history of head trauma, severe medical illness, and several serious mental disorders, including psychosis, bipolar disorder, substance abuse, conversion or factitious disorders, malingering, and suicidal or homicidal ideation.

Sociodemographic data and clinical characteristics were meticulously gathered. This included information on gender, age, educational background, employment status, marital status, domicile, smoking and alcohol consumption histories, prior medical conditions, and concurrent illnesses (refer to Table 2). Concerning primary symptoms, participants were subject to detailed interviews and completed a series of questionnaires. Our investigation encompassed a spectrum of symptoms, notably perceptions of self and environmental motion, sensations of lightheadedness, and instability. Additional symptom categories were explored. Furthermore, we evaluated factors exacerbating or alleviating symptoms, the duration and frequency of the disease and symptoms, as well as the clinical progression (as detailed in Table 2).

**TABLE 2:** Demographics of definite persistent postural‐perceptual dizziness (PPPD) and clinical variant of PPPD depending visual exaggerations.

		Total (*n* = 185)	Definite PPPD (*n* = 76, 41.1%) (a)	Clinical variant PPPD (*n* = 109, 58.9%)		*p* Value			
				cPPPD_visual pos (*n* = 21) (b)	cPPPD_visual neg (*n* = 88) (c)	(a) vs. (b + c)	(a) vs. (b)	(a) vs. (c)	(b) vs. (c)
**Age** (years), median (95% CI)		57 (55–59)	55.5 (52–58)	52 (46–59)	59 (57–61)	.186^M^	.051	.314	.027
**Gender** (male, %)		56 (30.3%)	19 (25%)	4 (19%)	33 (37.5%)	.193	.57	.086	.109
**Type of community**	Urban (*n*, %)	158 (85.9%)	67 (89%)	18 (85.7%)	73 (83%)	.263	.645	.244	.76
	Rural (*n*, %)	26 (14.1%)	8 (10.7%)	3 (14.3%)	15 (17%)				
**Marital status**	Married (*n*, %)	151 (81.6%)	63 (82.9%)	20 (95.2%)	68 (77.2%)	.709	.154	.371	.061
	Unmarried (*n*, %)	34 (18.4%)	13 (17.1%)	1 (4.8%)	20 (22.7)				
**Housing type**	Living with dependent person	146 (79.3%)	62 (82.7%)	1 (4.8%)	16 (18.2)	.46	.752	.363	.567
	Living with independent person	9 (4.9%)	2 (2.7%)	2 (9.5%)	6 (6.8%)				
	Alone	29 (15.8%)	11 (14.7%)	18 (85.7%)	66 (75%)				
**Professions**	Managers (*n*, %)	8 (4.3%)	3 (3.9%)	1 (4.8%)	4 (4.5%)	.261	.164	.158	.317
	Professionals and related workers (*n*, %)	21 (11.4%)	9 (11.8%)	3 (14.3%)	9 (10.2%)				
	Clerks (*n*, %)	21 (11.4%)	10 (13.2%)	5 (23.8%)	6 (6.8%)				
	Service workers (*n*, %)	19 (10.3%)	6 (7.9%)	0	13 (14.8%)				
	Sales workers (*n*, %)	8 (4.3%)	0	2 (9.5%)	6 (6.8%)				
	Agricultural, forestry, and fishery workers (*n*, %)	4 (2.2%)	2 (2.6%)	0	2 (2.3%)				
	Craft and related trades workers (*n*, %)	3 (1.6%)	0	0	3 (3.4%)				
	Equipment operators/products assemblers (*n*, %)	5 (2.7%)	3 (3.9%)	1 (4.8%)	1 (1.1%)				
	Elementary workers (*n*, %)	13 (7%)	7 (9.2%)	1 (4.8%)	5 (5.7%)				
	Housewives (*n*, %)	59 (31.9%)	23 (30.3%)	7 (33.3%)	29 (33%)				
	Jobless (*n*, %)	24 (13%)	13 (17.1%)	1 (4.8%)	10 (11.4%)				
**Education level**	Elementary school (6 years) (*n*, %)	19 (10.3%)	6 (7.9%)	1 (4.8%)	12 (13.6%)	.241	.525	.123	.067
	Middle school (9 years) (*n*, %)	22 (11.9%)	9 (11.8%)	4 (19%)	9 (10.2%)				
	High school (12 years) (*n*, %)	74 (40%)	27 (35.5%)	4 (19%)	43 (48.9%)				
	Associate degree (14 years) (*n*, %)	23 (12.4%)	14 (18.4%)	3 (14.3%)	6 (6.8%)				
	Bachelor's degree (16 years) (*n*, %)	35 (18.9%)	14 (18.4%)	7 (33.3%)	14 (15.9%)				
	Master's degree (18 years) (*n*, %)	10 (5.4%)	4 (5.3%)	2 (9.5%)	4 (4.5%)				
	Doctor of philosophy (21 years) (*n*, %)	2 (1.1%)	2 (2.6%)	0	0				
**History of smoking**	Non‐smoker (*n*, %)	138 (74.6%)	62 (81.6%)	2 (9.5%)	15 (17%)	.18	.966	.103	.459
	Ex‐smoker (*n*, %)	22 (11.9%)	6 (7.9%)	17 (81%)	59 (67%)				
	Current smoker (*n*, %)	25 (13.5%)	8 (10.5%)	2 (9.5%)	14 (15.9%)				
**Amount of alcohol**	Nondrinker (*n*, %)	131 (71.2%)	54 (71.1%)	9 (42.9%)	22 (25%)	.971	.226	.6	.11
	Drinker (*n*, %)	53 (28.8%)	22 (28.9%)	12 (57.1%)	65 (73.9%)				
**Coexisting conditions**	Hypertension (*n*, %)	51 (28.5%)	20 (27.4%)	1 (4.8%)	30 (34.1%)	.788	.028	.287	.006
	Diabetes mellitus (*n*, %)	22 (12.3%)	6 (8.2%)	1 (4.8%)	15 (17%)	.169	.595	.082	.14
	Hyperlipidemia (*n*, %)	52 (29.2%)	23 (31.9%)	2 (9.5%)	37 (30.7%)	.509	.041	.981	.041
	Cardiovascular disease (*n*, %)	11 (6.1%)	1 (1.4%)	1 (4.8%)	9 (10.2%)	**.027**	.342	.018	.413
	Migraine (*n*, %)	37 (20.7%)	13 (17.8%)	5 (23.8%)	19 (21.6%)	.433	.538	.479	.886
	Sleep disorder (insomnia) (*n*, %)	37 (20.7%)	19 (26%)	3 (14.3%)	15 (17%)	.142	.263	.201	.713
	Anxiety disorder (*n*, %)	21 (11.7%)	9 (12.3)	3 (14.3%)	9 (10.2%)	.837	.813	.731	.632
	Depressive disorder (*n*, %)	16 (8.9%)	9 (12.3%)	1 (4.8%)	6 (6.8%)	.187	.322	.26	.704
	Panic disorder (*n*, %)	6 (3.4%)	5 (6.8%)	1 (4.8%)	0	**.031**	.73	**.014**	.043
	Others (*n*, %)	21 (11.7%)	7 (8.2%)	3 (14.3%)	12 (13.6%)	.226	.405	.245	.984
	None (*n*, %)	39 (21.8%)	19 (26%)	6 (28.6%)	14 (15.9%)	.254	.816	.141	.204
**Primary symptoms (not mutually exclusive)**	Self‐motion (egomotion) (*n*, %)	104 (56.2%)	48 (63.2%)	14 (66.7%)	42 (47.7%)	.112	.767	.048	.119
	Lightheadedness (*n*, %)	64 (34.6%)	30 (39.5%)	6 (28.6%)	28 (31.8%)	.244	.36	.307	.773
	Unsteadiness (*n*, %)	60 (32.4%)	32 (42.1%)	5 (23.8%)	23 (26.1%)	**.019**	.127	.031	.826
	Surrounding motion (*n*, %)	42 (22.7%)	23 (30.3%)	3 (14.3%)	16 (18.2%)	**.040**	.143	.07	.672
	Not specified types (*n*, %)	37 (20%)	12 (15.8%)	2 (9.5%)	23 (26.1%)	.232	.47	.107	.104
**Precipitants (multiple choice)**	Benign paroxysmal positional vertigo (*n*, %)	70 (38.35)	28 (37.3%)	8 (38.1%)	34 (38.6%)	.831	.949	.82	.934
	Emotional stress (*n*, %)	60 (32.8%)	27 (36%)	9 (42.9%)	24 (27.3%)	.44	.566	.25	.173
	Vestibular migraine (*n*, %)	33 (18%)	12 (16%)	3 (14.3%)	18 (20.5%)	.551	.848	.444	.506
	Vestibular neuritis (*n*, %)	19 (10.4%)	10 (13.3%)	2 (9.5%)	7 (8%)	.275	.641	.274	.826
	Medical disease (*n*, %)	16 (8.7%)	6 (8%)	3 (14.3%)	7 (8%)	.767	.382	.991	.376
	Meniere's disease (*n*, %)	10 (5.5%)	6 (8%)	2 (9.5%)	2 (2.3%)	.209	.823	.095	.116
	Panic disorder (*n*, %)	10 (5.5%)	7 (9.3%)	2 (9.5%)	1 (1.1%)	.055	.979	.017	.036
	Trauma (*n*, %)	9 (4.9%)	4 (5.3%)	0	5 (5.7%)	.826	.28	.909	.261
	Others (*n*, %)	35 (19.1%)	16 (21.3%)	1 (4.8%)	18 (20.5%)	.527	.079	.92	.085
**Exacerbating factors**	Upright posture (*n*, %)	161 (87%)	76 (100%)	6 (28.6%)	79 (89.8%)	**<.001**	**<.01**	**.004**	**<.001**
	Active or passive motion (*n*, %)	168 (90.8%)	76 (100%)	13 (61.9%)	79 (89.8%)	**<.001**	**<.001**	**.004**	**.002**
	Exposure to moving/complex visual stimuli (*n*, %)	97 (52.4%)	76 (100%)	21 (100%)	0	**<.001**	n.a	n.a	n.a
**Exacerbating factors, details (multiple choice)**	Postural changes (*n*, %)	92 (52%)	43 (58.9%)	6 (28.6%)	43 (48.9%)	.112	.022	.333	.088
	Walking (*n*, %)	70 (39.5%)	35 (47.9%)	3 (14.3%)	32 (36.4%)	.056	.008	.213	.049
	Space‐motion discomfort (*n*, %)	39 (21.1%)	23 (30.3%)	5 (23.8%)	11 (12.5%)	**.011**	.563	**.005**	.188
	Watching television (*n*, %)	26 (14.7%)	20 (27.4%)	5 (23.8%)	1 (1.1%)	**<.001**	.83	**<.001**	**<.001**
	Riding in a transportation (*n*, %)	44 (24.9%)	28 (38.4%)	5 (23.8%)	11 (12.5%)	**<.001**	.269	**<.001**	.185
	Sitting without back or arm support (*n*, %)	24 (13.6%)	17 (23.3%)	1 (4.8%)	6 (6.8%)	**.002**	.067	**.004**	.731
	Using a computer or smartphone (*n*, %)	35 (19.8%)	27 (37%)	7 (33.3%)	1 (1.1%)	**<.001**	.87	**<.001**	**<.001**
	Reading small letters (*n*, %)	40 (22.6%)	30 (41.1%)	9 (42.9%)	1 (1.1%)	**<.001**	.754	**<.001**	**<.001**
	Standing without support (*n*, %)	36 (20.3%)	21 (28.8%)	1 (4.8%)	14 (15.9%)	**.02**	.027	.069	.182
	Housework or light exercises (*n*, %)	69 (39%)	37 (50.7%)	1 (4.8%)	31 (35.2%)	**.007**	**<.001**	.082	**.005**
	Head moving (*n*, %)	94 (53.1%)	54 (74%)	7 (33.3%)	33 (37.5%)	**<.001**	**.001**	**<.001**	.723
	Sleep deprivation (*n*, %)	72 (40.7%)	35 (47.9%)	10 (47.6%)	27 (30.7%)	.099	.871	.043	.134
	Emotional stress (*n*, %)	74 (41.8%)	34 (46.6%)	10 (47.6%)	30 (34.1%)	.281	.786	.167	.238
	Overwork (*n*, %)	41 (23.2%)	23 (31.5%)	3 (14.3%)	15 (17%)	**.028**	.145	.046	.761
	Others (*n*, %)	14 (7.9%)	4 (5.5%)	1 (4.8%)	9 (10.2%)	.316	.933	.235	.436
**Relieving factors (multiple choice)**	Lying down (*n*, %)	105 (57.1%)	47 (61.8%)	4 (19%)	54 (61.4%)	.272	.001	.976	<.001
	Resting (*n*, %)	76 (41.3%)	43 (56.6%)	0	33 (37.5%)	**<.001**	<.001	**.017**	**.001**
	Light conversation (*n*, %)	37 (20.1%)	13 (17.1%)	8 (38.1%)	16 (18.2%)	.394	.039	.83	.051
	Others (*n*, %)	29 (15.8)	8 (10.5%)	9 (42.9%)	12 (13.6%)	.102	.001	.526	.003
**Disease duration** (months after symptom onset)		12 (8–12)	12 (9–24)	8 (4–24)	10.5 (5–12)	**.008^M^**	**.008^M^**	.203^M^	.628^M^
**Clinical course**	No change (*n*, %)	91 (49.5%)	35 (46.1%)	12 (57.1%)	44 (50%)	.201	.675	.26	.851
	Relieved (*n*, %)	57 (31%)	21 (27.6%)	6 (28.6%)	30 (34.1%)				
	Gradually worsening (*n*, %)	35 (19%)	19 (25%)	3 (14.3%)	13 (14.8%)				
**Time/day** (hours)		5 (2.5–10)	5 (3–10)	2 (1–24)	4 (2–10)	.793^M^	.798^M^	.886^M^	.936^M^
**Days/week**		7 (7–7)	7 (7–7)	7 (2–7)	7 (6–7)	.474^M^	.674^M^	.255^M^	.349^M^

*Note*: Values are expressed as median (95%confidence interval‐CI) or number (percentage). Statistical significance was calculated using Chi‐square test or Mann–Whitney *U* test. Bold values denote a statistically significant difference.

This study was reviewed and approved by the Institutional Review Board at each participating center, including the Jeonbuk National University Hospital (IRB 2020‐06‐033). The patients provided written informed consent to participate in this study and for the publication of any potentially identifiable data included in this article.

### 2.2 Dizziness and balance functional assessment

*The dizziness handicap inventory (DHI)* is a self‐assessment instrument commonly employed to measure the influence of balance problems and dizziness on daily activities. The questionnaire comprises 25 items that are categorized into three areas: physical (7), emotional (9), and functional (9) domains. Each question is rated on a 4‐point Likert scale ranging from 0 (no difficulty) to 4 (extreme difficulty) (Han et al., 2004; Jacobson & Newman, 1990).

### 2.3 Sleep disorder assessment

*The pittsburgh sleep quality index (PSQI)* is a tool that evaluates seven aspects related to sleep, including sleep quality, duration, latency, habitual sleep efficiency, sleep disturbances, daytime dysfunction, and use of sleep medication. Each component can be scored from 0 to 3 points, and the total possible score is 21 points (Sohn et al., 2012).

### 2.4 Anxiety‐related personality assessments

*The patient health questionnaire‐9 (PHQ‐9)* is a self‐report instrument commonly used to screen for depression. It includes nine questions that evaluate the frequency and intensity of symptoms experienced in the past 2 weeks, and each question is rated on a 0–3 scale (Han et al., 2008; Huang et al., 2006; Zhang et al., 2022).

*The state‐trait anxiety inventory X1 (STAI‐X1)* is a standardized psychological assessment tool that measures the level of anxiety in individuals. The scale consists of 20 items, each rated on a 4‐point Likert scale from 1 (not at all) to 4 (very much) (Czorniej et al., 2022; Kim & Shin, 1978).

*The big five inventory (BFI‐K)* is an assessment tool commonly used to evaluate an individual's personality traits based on five dimensions, namely, Extraversion, Agreeableness, Conscientiousness, Neuroticism, and Openness to Experience. The inventory includes 44 questions, with 8 items for each trait except Openness, which has 10 items (Goldberg, 1993; Kim et al., 2010). Participants rate their level of agreement with each item on a 5‐point scale ranging from 1 (strongly disagree) to 5 (strongly agree).

*The general self‐efficacy scale (GSE)* is a psychometric scale used to measure an individual's optimistic self‐beliefs in coping with challenging demands. The scale comprises 10 items, and each item is rated on a 4‐point Likert scale, ranging from 1 (not true at all) to 4 (exactly true) (Indovina et al., 2019).

*The brief resilience scale (BRS)* is a self‐reported measure designed to gauge an individual's ability to cope with stress and adversity. It consists of six items, each asking respondents to rate their level of agreement on a 5‐point Likert scale (ranging from 1 for complete disagreement to 5 for complete agreement) with statements about their ability to recover from setbacks (Chmitorz et al., 2018; Kwon & Kwon, 2014; Smith et al., 2008).

We included these measures to provide a more comprehensive understanding of the PPPD patient experience and to explore potential interactions between PPPD and these psychological and sleep‐related factors. The inclusion of these measures acknowledges the complex, multifaceted nature of PPPD, where psychological well‐being and sleep quality can play a crucial role in the severity and management of the condition.

### 2.5 Statistical analysis

The data were analyzed using SPSS Statistics version 23.0 (IBM Corp.) and various statistical tests were used based on the nature of the variables. Parametric data were analyzed with a one‐way analysis of variance and nonparametric data were analyzed using the Mann–Whitney *U* test. The statistical significance level was determined as *p* < .05, and for the post hoc test (LSD test), a Bonferroni‐adjusted significance level of *p* < .017 (0.05/3) was applied.

## 3 RESULTS

### 3.1 Sociodemographic and clinical data

A total of 185 participants were consecutively enrolled in the study, consisting of 56 males and 129 females, with an average age of 54.8 ± 13.5 years (Table 2). The majority of patients, comprising 85.9% (158/185), resided in urban areas. Moreover, a relatively high level of education was observed among the patients, with 77.8% (144/185) having completed their education beyond high school. Hyperlipidemia was the most common coexisting condition, with a prevalence of 29.2%, followed by hypertension at 28.5%, and migraine and sleep disorders at 20.7% each. The mean disease duration was 12 months (95% CI: 8–12), and dizziness symptoms occurred daily, lasting an average of 5 h per day (95% CI: 2.5–10). In terms of clinical progression, 49.5% of the patients experienced no change in their condition. Meanwhile, 31% felt some relief but still faced substantial functional challenges in their day‐to‐day activities. Additionally, 19% noted a gradual deterioration in their condition. For primary symptoms, self‐motion was the most common (56.2%), and followed by lightheadedness (34.6%), unsteadiness (32.4%), and surrounding motion (22.7%). Regarding PPPD precipitants, benign paroxysmal positional vertigo (BPPV) was the most common, reported by 38.35% of patients, followed by emotional stress at 32.8%, vestibular migraine at 18%, vestibular neuritis at 10.4%, medical disease at 8.7%, and other conditions such as Meniere's disease, panic disorder, and trauma accounting for approximately 5% (Habs et al., 2020).

Criterion B exacerbating factors included active or passive motion reported by 90.8% of patients, upright posture at 87%, and exposure to moving or complex visual stimuli at 52.4%. Further breakdown showed that persistent dizziness symptoms were aggravated by head movement in 53.1% of patients, postural changes in 52%, emotional stress in 41.8%, sleep deprivation in 40.7%, walking in 39.5%, housework or light exercises in 39%, riding in transportation in 24.9%, overwork in 23.2%, reading small letters in 22.6%, and standing without support in 20.3%. Patients reported that lying down (57.1%), resting (41.3%), engaging in light conversation (20.1%), or other undefined factors (15.8%) alleviated their symptoms of dizziness.

**FIGURE 2:**
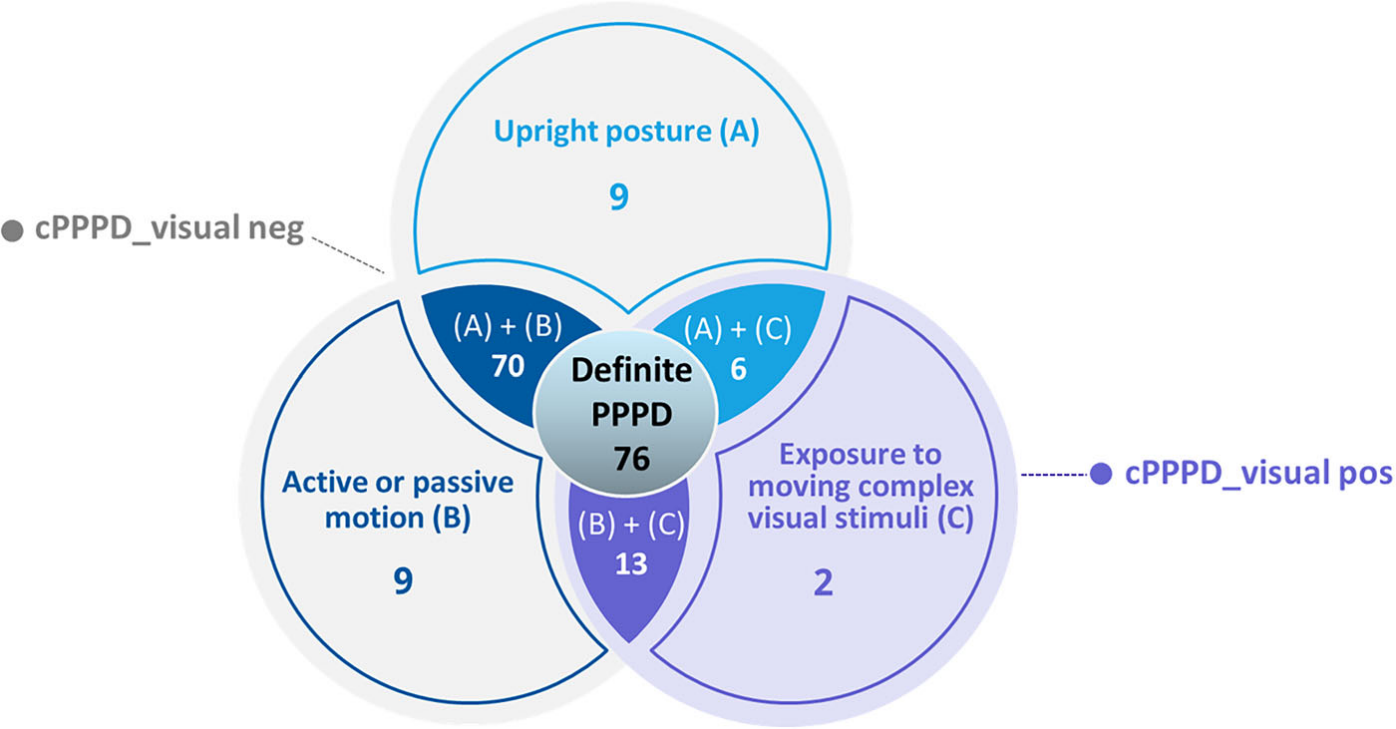
Venn diagram illustrating the distribution of clinical variant persistent postural‐perceptual dizziness (PPPD) patients based on visual exacerbation.

Among the clinical variant PPPD group (*n* = 109), the occurrence of each exacerbating factor was determined to be active or passive motion in 84.4% (factor A, *n* = 92), upright posture in 78% (factor B, *n* = 85), and exposure to moving or complex visual stimuli in 19.3% (factor C, *n* = 21), respectively (Figure 2). The data suggest that the majority of patients with clinical variant PPPD displayed two exacerbating factors (81.65%, *n* = 89), with the most prevalent combination being factors A + B (64.22%), followed by B+C (11.93%), and A + C (5.5%). Among patients with only one exacerbating factor (18.35%, *n* = 20), factors A and B were equally frequent, each being present in 8.26% (*n* = 9) of patients. Visual stimuli (factor C) was the least common exacerbating factor overall, only found in 1.83% (*n* = 2) of patients (Figure 2).

Regarding the assessment of unsteadiness characteristics and personalities, the study used several scales to measure different factors (Table 3). The DHI had a total score of 39.97 ± 23.37, indicating a moderate level of handicap. The DHI was further divided into three sub‐scores: DHI‐functional, DHI‐emotional, and DHI‐physical, with scores of 16.27 ± 10.48, 13.88 ± 9.46, and 9.83 ± 6.16, respectively. The PSQI had a global score of 9.11 ± 4.2, indicating a moderate level of sleep difficulty. The PHQ‐9 score was 8.47 ± 5.94, indicating a moderate level of depression, whereas the score for STAI‐X1 was 44.86 ± 8.71, which falls within the normal range for anxiety. The study also measured personality traits using the BFI, which yielded the following sub‐scores: Extraversion at 3.03 ± 0.68, Agreeableness at 2.97 ± 0.71, Conscientiousness at 3.14 ± 0.71, Neuroticism at 2.88 ± 0.64, and Openness at 2.86 ± 0.74. Lastly, the GSE yielded a score of 27.91 ± 4.96, indicating a high level of self‐efficacy, whereas the BRS yielded a score of 17.69 ± 4.42, indicating a low level of resilience.

**TABLE 3:** Dizziness impact and neuropsychiatric personality traits in definite persistent postural‐perceptual dizziness (PPPD) and clinical variant with and without visual aggravation.

Questionnaire		Definite PPPD (*n* = 76) (a)		Clinical variant PPPD (*n* = 109)					*p*‐Value		
				cPPPD_visual pos (*n* = 21) (b)		cPPPD_visual neg (*n* = 88) (c)					
		Mean	SD	Mean	SD	Mean	SD	ANOVA	Post hoc (LSD) test		
									(a) vs. (b)	(a) vs. (c)	(b) vs. (c)
**DHI**	DHI‐functional	19.11	11.21	14.86	10.15	14	9.56	**.002**	.061	**.001**	.739
	DHI‐emotional	16.26	10.54	14.48	9.16	12.47	9.67	.102	.614	.034	.387
	DHI‐physical	11.58	5.53	8.6	5.55	8	6.24	**.001**	**.009**	**.001**	.681
	Total DHI	46.95	24.69	37.33	22.11	35.07	2.39	**.018**	.163	**.005**	.688
**PSQI**	Subjective sleep quality	1.57	0.76	1.5	0.69	1.23	0.86	**.004**	.426	**.001**	.183
	Sleep latency	2.01	1.05	1.57	0.99	1.35	1.06	**.001**	**.004**	**.001**	.393
	Sleep duration	1.15	1.19	1.05	1.1	0.94	1.18	.614	.802	.326	.707
	Habitual sleep efficiency	0.96	1.16	0.6	0.82	0.65	0.96	.062	.855	.029	.349
	Sleep disturbances	1.49	0.67	1.4	0.68	1.53	0.78	.516	.263	.531	.461
	Use of sleep medication	1.24	1.27	1.15	1.23	1.07	1.09	.616	.722	.326	.788
	Daytime dysfunction	1.58	0.99	1.4	0.94	1.36	0.97	.171	.31	.067	.884
	PSQI global score	9.75	4.22	8.45	3.36	8.33	4.09	**.005**	.059	**.002**	.902
**PHQ‐9**		9.58	6.33	7.36	4.71	6.75	5.41	**.004**	.02	**.003**	.674
**STAI‐X1**		44.59	8.34	43.5	8.3	44.86	8.86	.735	.434	.792	.532
**BFI‐K**	Extraversion	3.14	0.47	23.95	5.21	3.6	0.39	.453	.24	.418	.5
	Agreeableness	3.06	0.55	27.05	5.68	26.99	4.49	.564	.548	.302	.961
	Conscientiousness	3.29	0.53	28.15	6	28.67	4.45	.59	.368	.446	.67
	Neuroticism	3.01	0.46	23.15	4.64	23.02	3.41	.148	.287	.058	.894
	Openness	2.97	0.59	29.05	6.77	28.71	5.59	.499	.572	.21	.821
**GSE**		29.03	4.46	28.7	3.54	27.08	4.95	.102	.964	.045	.188
**BRS**		17.17	4.67	18.05	4.27	18.27	3.88	.148	.056	.314	.842

*Note*: Values are expressed as mean (SD). Statistical significance was calculated using one‐way ANOVA with LSD (least significant difference) post hoc test. The statistical significance level for ANOVA was set at *p* < .05, whereas for the post hoc test (LSD test), a Bonferroni‐adjusted significance level of *p* < .017 (0.05/3) was used. Bold values denote a statistically significant difference. Clinical variant PPPD, with or without visual exacerbation, was defined using the Niigata PPPD questionnaire (NPQ), with questions 2, 4, 8, and 10. A cutoff value of 9 out of a total score of 24 was used: NPQ ≤ 9 indicated PPPD without visual exacerbation, whereas NPQ > 9 indicated PPPD with visual exacerbation.

Abbreviations: ANOVA, analysis of variance; BFI‐K, big five inventory; BRS, brief resilience scale; DHI, dizziness handicap inventory; GSE, general self‐efficacy scale; PSQI, pittsburgh sleep quality index; PHQ‐9, patient health questionnaire‐9; STAI‐X1, state‐trait anxiety inventory X1.

### 3.2 Comparison between groups with definite PPPD and clinical variant of PPPD

In the study, 76 patients were diagnosed with definite PPPD, whereas 109 patients were diagnosed with clinical variant PPPD. Table 2 indicates that there were no significant differences in demographic characteristics between the definite PPPD and clinical variant PPPD groups. Our analysis differentiates the clinical variant of PPPD from definitive PPPD in several key aspects. For core symptoms (Criterion A, Table 2), the definite PPPD group exhibited a higher frequency of unsteadiness (42.1% vs. 25.7%, *p* = .019, Chi‐square test) and perception of surrounding motion (30.3% vs. 17.4%, *p* = .04, Chi‐square test) compared to the clinical variant group. Regarding sensitivity to exacerbating factors (Criterion B, detailed in Table 2), patients with definitive PPPD were significantly more affected by activities such as watching television, using a computer or smartphone, reading small letters, riding in transportation, moving their head, sitting without back or arm support, engaging in housework or light exercises, standing without support, and overwork, in comparison to the clinical variant group. In terms of precipitants (Criterion C, Table 2), no notable differences were observed between the groups. However, clinical dizziness and neuropsychiatric scales showed that the definite PPPD group had significantly higher scores on the DHI (46.95 ± 24.69 vs. 35.06 ± 21.16, *p* = .001, independent samples *t*‐test) and its sub‐scores (DHI‐functional, 19.11 ± 11.21 vs. 14.28 ± 9.49, *p* = .02; DHI‐emotional, 16.26 ± 10.54 vs. 12.19 ± 8.26, *p* = .04; DHI‐physical, 19.11 ± 5.53 vs. 14.28 ± 6.31, *p* = .01, independent samples *t*‐test), as well as on the PSQI sub‐score for sleep latency (2.01 ± 1.05 vs. 1.6 ± 1.06, *p* = .011, independent samples *t*‐test), PHQ‐9 (9.58 ± 6.33 vs. 7.66 ± 5.54, *p* = .032), and GSE (29.03 ± 4.46 vs. 27.11 ± 5.17, *p* = .01) in comparison to the clinical variant group, representing quantitative measures of Criterion D (Table 3). Further, analysis of coexisting conditions revealed that the definite PPPD group had a significantly higher prevalence of panic disorder (6.8% vs. 0.9%, *p* = .031, Chi‐square test) and a longer duration of disease (12 vs. 10 months, *p* = .008, Mann–Whitney *U* test).

### 3.3 Sub‐analysis of clinical variant PPPD with and without visual aggravation

Among the three aggravating factors, the visual factor exhibited the most pronounced difference between definitive PPPD and the clinical variant of PPPD, suggesting it possesses significant diagnostic strength in PPPD. For visual stimuli, the AUC was 0.83 with a sensitivity of 82% and specificity of 74%. In comparison, for motion stimuli, the AUC was 0.684, sensitivity stood at 60%, and specificity at 70%. For upright stimuli, the AUC was 0.723, with a sensitivity of 76% and specificity of 60%.

When comparing between the definite PPPD and clinical variant PPPD with visual exacerbations (cPPPD_visual pos), there were no significant differences in sociodemographic characteristics, primary symptoms, clinical course, duration of daily dizziness, weekly frequency of dizziness, and specific precipitants between both groups. Only disease duration was significantly longer in definite PPPD compared with variant with visual exaggeration. Additionally, no significant differences were observed in the personality traits and anxiety scales between the two groups. However, significant differences were observed in two subscales in the DHI‐physical (*p* = .009) and PSQI‐sleep latency (*p* = .004) (Table 3). Conversely, significant disparities emerged in multiple parameters when comparing the definite PPPD and clinical variant PPPD groups without visual exacerbations (cPPPD_visual neg). These included the DHI‐total score (*p* = .005) and subscales (functional: *p* = .001, physical: *p* = .001), PSQI‐global score (*p* = .002) and subscales (subjective sleep quality: *p* = .001; sleep latency: *p* = .001), along with PHQ‐9 (*p* = .003). The variant without visual exaggeration also displayed significantly reduced rates of panic disorder, diminished space‐motion discomfort, lesser impact of dizziness, and decreased prevalence of depression when compared with the definitive PPPD (Table 3).

Meanwhile, when comparing clinical parameters between the cPPPD_visual pos and cPPPD_visual neg subgroups, no significant differences were noted in balance scales and personality traits. Likewise, with regards to demographic and clinical characteristics, and the precipitants of PPPD, the data revealed no notable difference between the two categories. In lines with definite PPPD, in both variant groups, BPPV emerged as the most prevalent precipitant (38.9%), succeeded by emotional stress (30.6%), vestibular migraine (19.4%), and vestibular neuritis (8.3%) (Table 3).

## 4 DISCUSSION

This study examined the sociodemographic and clinical characteristics of Korean patients with PPPD, including variants that did not meet all exaggerating criteria, which had not been previously investigated (Das et al., 2023; Popkirov, Staab, et al., 2018; Staab, 2020). The participants had an average age in the mid‐50s, and the majority were female, consistent with recent research (Adamec et al., 2020; Bittar & Lins, 2015; Sarna et al., 2021; Trinidade & Goebel, 2018; Wang et al., 2021; Waterston et al., 2021; Yan et al., 2017; Zhang et al., 2022). The higher prevalence of PPPD in women may be attributed to their increased likelihood of experiencing certain vestibular disorders such as vestibular migraine or BPPV (Becker‐Bense et al., 2019; Neuhauser, 2016; Yetiser & Ince, 2015), as well as their increased likelihood of functional neurological disorders (Häuser et al., 2013).

The study found that the most common primary symptoms of PPPD were self‐motion (56.2%), lightheadedness (34.6%), unsteadiness (32.4%), and surrounding motion (22.7%). BPPV was the most common precipitating condition (38.35%), followed by emotional stress (32.8%), vestibular migraine (18%), and vestibular neuritis (Dieterich & Staab, 2017; Popkirov, Staab, et al., 2018; Staab et al., 2017). In this study's questionnaire findings, Korean patients with PPPD exhibited moderate dizziness severity, accompanied by notable instances of poor sleep quality and depression. These patients demonstrated high self‐efficacy, yet their resilience levels were comparatively low. Notably, their levels of introversion and neuroticism did not significantly differ from those of the general population. Resilience, defined as the ability to effectively navigate and adapt to stressors and adverse situations, including mental or physical health challenges, is a critical factor in managing chronic conditions (Smith et al., 2008). The juxtaposition of high self‐efficacy with low resilience might lead to frustration, especially under circumstances that challenge an individual's control, such as specific triggering events for dizziness. This interplay potentially exacerbates chronic dizziness by adversely affecting depression and anxiety levels. Aligning with prior studies, a higher incidence of depression was observed among PPPD patients in this cohort (Bittar & Lins, 2015; Staab et al., 2017; Thompson et al., 2015). However, their anxiety scores, as measured by the STAI‐X1 questionnaire, were not particularly high, although a more stringent scoring criteria revealed moderate to high levels of anxiety (Kayikcioglu et al., 2017). Previous research has suggested that neuroticism and anxiety‐related personality traits may be precursors to PPPD, as they can lead to a hypervigilant state of increased introspective self‐monitoring in response to fear of further vertigo attacks (Chiarella et al., 2016; Yan et al., 2017; Yu et al., 2018). However, patients in this study did not exhibit high levels of neuroticism, as measured by the BFI‐K personality inventory (Staab et al., 2014). These findings are significant and bolster recent shifts in understanding, moving away from viewing PPPD as a mere psychosomatic manifestation of anxiety to long‐term functional neurological disorder in individuals who have experienced acute illnesses with vestibular symptoms (Staab, 2023). Due to these considerations, thus posing challenges in diagnosing patients who exhibit symptoms of chronic functional dizziness that align with PPPD but do not meet all the diagnostic criteria. Alternatively, these variations could be attributed to differences in Asian PPPD patients, a perspective that necessitates further investigation.

In our study, we observed that a significant portion, 59% (109 out of 185 patients), exhibited only one or two exacerbating factors, as specified in Criterion B, a subthreshold variant of PPPD. This observation supports the need to validate of considering a subthreshold variant as a distinct clinical entity. Recognizing this variant is crucial as it suggests that a notable number of patients with severe chronic vestibular symptoms, who would otherwise be without a diagnosis, may actually fall within this subthreshold category. This has important implications for both diagnosis and therapeutic approaches. A prior study reported that roughly half of the patients experienced one or two out of the three types of exacerbating factors within the first 90 days of vestibular symptom onset. However, during subsequent monitoring, approximately 10% of these patients developed PPPD, suggesting that these individuals were prone to its exacerbating factors early on after the symptoms appeared (Kabaya et al., 2022). This consistency underscores the potential stability of the subthreshold PPPD variant as a clinical condition, rather than being merely a preliminary stage of full PPPD. PPPD could be diverse in nature, with possible different subtypes, and atypical visuo‐vestibular processing could make some people more susceptible to visually induced dizziness (Powell et al., 2020). This susceptibility could be worsened by a vestibular insult or a more generalized insult. Due to these considerations, thus posing challenges in diagnosing patients who exhibit symptoms of functional dizziness that align with PPPD but do not meet all the diagnostic criteria (Hüfner & Sperner‐Unterweger, 2019). The present study compares the differences between groups with definite and clinical variant PPPD, revealing no significant differences in sociodemographics, clinical course, duration and frequency of dizziness, and specific precipitants between the two groups. However, the definite PPPD group showed significantly higher rates of panic disorder, longer disease duration, and greater frequencies of unsteadiness, surrounding motion, and space‐motion discomfort compared to the clinical variant PPPD group (Criterion A). Additionally, the definite PPPD patients were more sensitive to variable exacerbating factors such as watching television or using a computer or smartphone (Criterion B). Although there were no notable differences were observed in the precipitants (Criterion C), the definite PPPD group also scored higher on balance scales and personality tests, including DHI, sleep latency of PSQI, PHQ‐9, and GSE (Criterion D). As the definite PPPD group had a significantly longer disease duration in clinical manifestation, it is possible that the clinical variant PPPD group may progress to the definite group over time.

In the present study, an effort was also made to divide the clinical variant of PPPD into two categories: cPPPD_visual pos and cPPPD_visual neg, based on items representing the visual factor of the NPQ, consistent with a PPPD model proposed in recent studies (Yagi et al., 2021). The findings revealed that definite PPPD presented more pronounced differences in balance scales and personality tests compared with cPPPD_visual neg, but not cPPPD_visual pos. This implies that the clinical variant of PPPD with visual exacerbation may share common traits and similarities with definite PPPD, and that the clinical variant of PPPD without visual exacerbation might represent a separate disease entity. Based on these findings, it is tentatively suggested that the visual component of Criterion B could be a pivotal component amplifying the diagnosis of PPPD. In our examination of the three aggravating factors for PPPD, the visual factor displayed the most significant difference between definitive PPPD and its clinical variants. A variant of PPPD without visual aggravation may indicate a distinct disease entity with milder symptoms. Thus, those experiencing visual exacerbation but not meeting all the requirements of diagnostic Criterion B might be categorized either as a subcategory with a visual‐dominant subtype or recognized as a clinical variant of PPPD. However, while there may be differences in how visual and non‐visual symptoms manifest in PPPD, these variations alone might not be sufficient to classify them as separate subtypes or distinct disorders. Further research and more comprehensive analysis, possibly involving larger sample sizes or additional variables, may be required to clarify the relationship between these groups and to understand the full spectrum of PPPD manifestations (Yagi et al., 2021).

The limitation of this study, with regard to the selective recruitment of patients and the exclusion of those with active neuro‐otologic disorders, “organic” brain diseases, and head trauma, lies primarily in its impact on the generalizability of the findings. By selecting a specific subset of patients for the study, the results may not accurately represent the broader PPPD patient population, which often includes individuals with these coexisting conditions. This selective approach, while useful for reducing confounding variables and enabling a more focused analysis of PPPD symptoms, means that the study's findings may not be directly applicable to all PPPD patients, especially those whose condition coexists with other neuro‐otologic or brain disorders. Consequently, the conclusions drawn from this study should be interpreted with caution when considering the broader PPPD patient population. The study's insights might be more relevant to a specific group of PPPD patients who do not have these excluded conditions, thereby limiting the scope of its applicability.

## AUTHOR CONTRIBUTIONS

**Joo Hyun Park**: Investigation; methodology; writing—original draft; data curation; formal analysis; supervision. **Thanh Tin Nguyen**: Investigation; writing—original draft; methodology; visualization; data curation; formal analysis. **Sung‐Hee Kim**: Investigation; methodology; formal analysis; data curation. **Ji‐Yun Park; Seunghee Na; Eun‐Ju Jeon; Ji won Seo; Chang Gun Cho; Se‐Joon Oh; Sung‐Won Choi; Kwang‐Dong Choi; Seo‐Young Choi; Ji Eun Choi; Sung‐Kwang Hong; Won‐Ho Chung; Young Sang Cho; Hwan Ho Lee; Yong‐Hwi An; Kyu‐Hee Han**: Methodology; data curation. **Hyung Lee**: Data curation; methodology; resources; project administration. **Hyun Ah Kim; Ho Yun Lee; Jong‐Dae Lee; Se A Lee**: Methodology; data curation. **Sun‐Young Oh**: Conceptualization; funding acquisition; writing—original draft; writing—review and editing; visualization; supervision; resources; validation.

## CONFLICT OF INTEREST STATEMENT

The authors declare that the research was conducted in the absence of any commercial or financial relationships that could be construed as a potential conflict of interest.

### PEER REVIEW

The peer review history for this article is available at https://publons.com/publon/10.1002/brb3.3389.

## Data Availability

All individual data of the participants that underlie the results reported in this article, after de‐identification (manuscript, tables, and figures), will be shared.
